# Genetic stability of Rift Valley fever virus MP-12 vaccine during serial passages in culture cells

**DOI:** 10.1038/s41541-017-0021-9

**Published:** 2017-07-17

**Authors:** Nandadeva Lokugamage, Tetsuro Ikegami

**Affiliations:** 1 Department of Pathology, The University of Texas Medical Branch at Galveston, 301 University Blvd., Galveston, TX 77555 USA; 2 The Sealy Center for Vaccine Development, The University of Texas Medical Branch at Galveston, 301 University Blvd., Galveston, TX 77555 USA; 3 The Center for Biodefense and Emerging Infectious Diseases, The University of Texas Medical Branch at Galveston, 301 University Blvd., Galveston, TX 77555 USA

## Abstract

Rift Valley fever is a mosquito-borne zoonotic disease endemic to Africa, which affects both ruminants and humans. Rift Valley fever causes serious damage to the livestock industry and is also a threat to public health. The Rift Valley fever virus has a segmented negative-stranded RNA genome consisting of Large (L)-segment, Medium (M)-segment, and Small (S)-segment. The live-attenuated MP-12 vaccine is immunogenic in livestock and humans, and is conditionally licensed for veterinary use in the US. The MP-12 strain encodes 23 mutations (nine amino acid substitutions) and is attenuated through a combination of mutations in the L-segment, M-segment, and S-segment. Among them, the M-U795C, M-A3564G, and L-G3104A mutations contribute to viral attenuation through the L-segment and M-segment. The M-U795C, M-A3564G, L-U533C, and L-G3750A mutations are also independently responsible for temperature-sensitive phenotype. We hypothesized that a serial passage of the MP-12 vaccine in culture cells causes reversions of the MP-12 genome. The MP-12 vaccine and recombinant rMP12-ΔNSs16/198 were serially passaged 25 times. Droplet digital polymerase chain reaction analysis revealed that the reversion occurred at L-G3750A during passages of MP-12 in Vero or MRC-5 cells. The reversion also occurred at M-A3564G and L-U533C of rMP12-ΔNSs16/198 in Vero cells. Reversion mutations were not found in MP-12 or the variant, rMP12-TOSNSs, in the brains of mice with encephalitis. This study characterized genetic stability of the MP-12 vaccine and the potential risk of reversion mutation at the L-G3750A temperature-sensitive mutation after excessive viral passages in culture cells.

## Rift Valley fever: Whole-virus vaccine alters the attenuation profile

A vaccine candidate for Rift Valley fever virus can undergo mutations and partially revert to parental pathogenic strain. Rift Valley fever is a prolific infection that affects livestock and humans in Africa. Tetsuro Ikegami and Nandadeva Lokugamage, of the University of Texas Medical Branch, United States, tested the genetic stability of vaccine candidate MP-12, an attenuated form of the virus, through ‘serial passaging’—culturing the virus, establishing a new culture from a sample, then repeating the procedure to determine how the virus mutates in response to successive new environments. The duo found that after 25 ‘passages’, MP-12 reverted a part of attenuation mutations back to its original, pathogenic sequence. Though MP-12 redundantly encodes stable attenuation mutations, it will be important to use a seed lot system to avoid the attenuation profile changes.

## Introduction

Rift Valley fever virus (RVFV) is a high consequence zoonotic pathogen that is classified as a Category A priority pathogen by the National Institute of Health (NIH), and an overlap select agent by the US Department of Health and Human Service and the US.Department of Agriculture (USDA).^1–3^ Rift Valley fever (RVF) is endemic to sub-Saharan Africa, but has spread into Egypt, Madagascar, Saudi Arabia, and Yemen.^4–6^ RVFV is transmitted by mosquitoes, and causes high rates of abortion and fetal malformation in pregnant ruminants.^7, 8^ Transmission of RVFV to humans occurs through exposure to the bodily fluids of infected animals, or from the bite of an infected mosquito.^8^ The majority of RVF patients have self-limiting febrile illness or are asymptomatic, and the estimated mortality rate is less than 3.0%.^9^ In contrast, higher mortality rates have been reported among hospitalized RVF patients with severe clinical manifestations, such as hemorrhage, jaundice, neurological disorders, or blindness.^10, 11^ Vaccination is an effective measure by which to prevent RVFV spread, both in endemic and non-endemic countries. In the US, a formalin-inactivated RVF vaccine (TSI-GSD-200) and the live-attenuated MP-12 vaccine are investigational new drugs, and have been tested in human clinical trials.^12–15^ The MP-12 vaccine received a conditional license for veterinary use from the USDA since 2013, and the master-seed stock has been generated by Zoetis, Inc.^16^ Vaccine lots of the MP-12 vaccine must be prepared on a large scale in case of RVF outbreak^16^; therefore, an understanding of the genetic stability of the MP-12 vaccine during serial viral passages in culture cells is important.

**Fig. 1:**
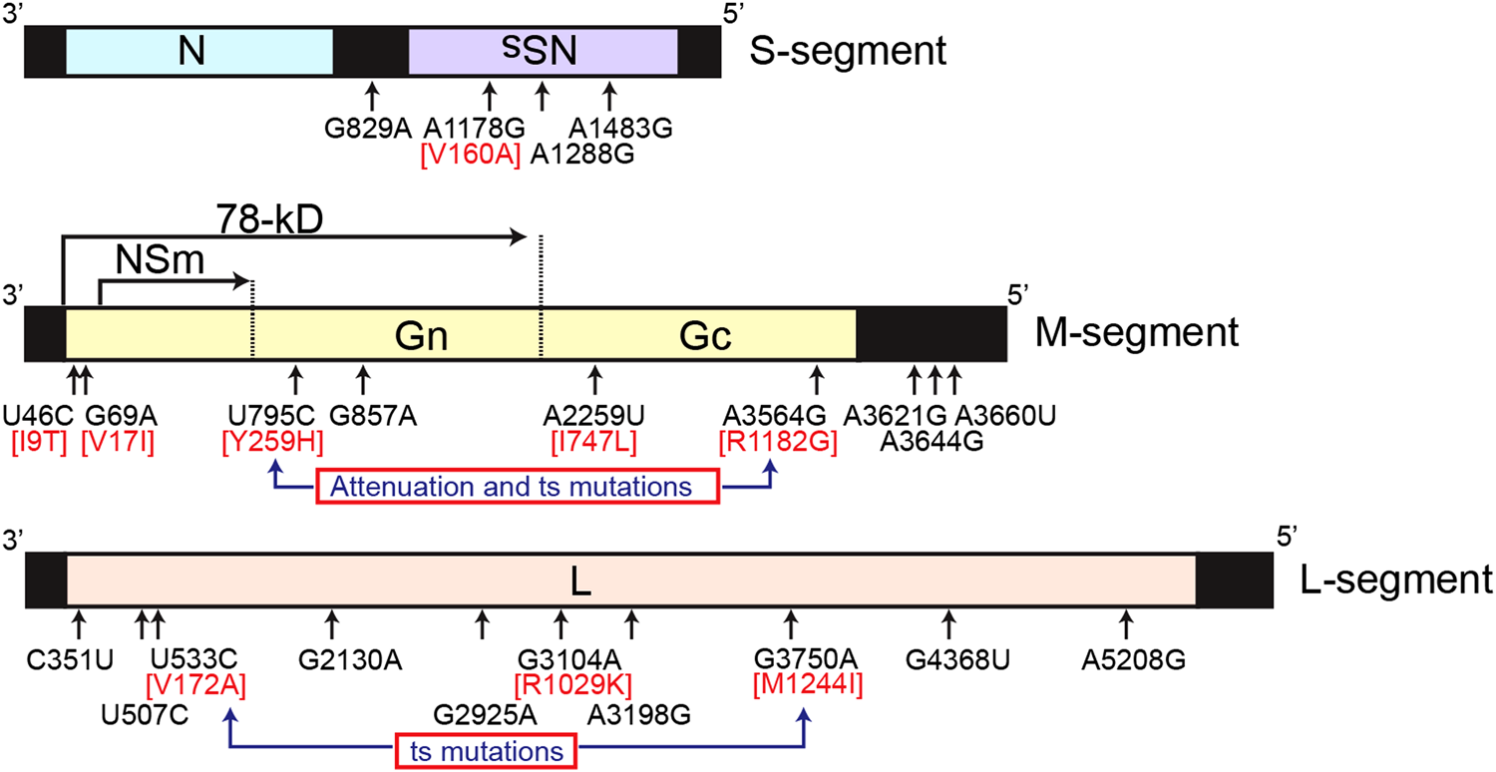
Schematics of MP-12 vaccine mutations. The genomic RNA of Small (S)-segment, Medium (M)-segment, and Large (L)-segment are shown. Mutations of the MP-12 vaccine compared to the parental ZH548 strain are indicated by *arrows*, and amino acid substitutions are shown in *red*. Reported attenuation mutations in the M-segment and temperature-sensitive (ts) mutations in the M-segments and L-segments are also shown

RVFV belongs to the genus *Phlebovirus* in the family *Bunyaviridae*, and its negative-stranded RNA genome is comprised of Large (L)-segment, Medium (M)-segment, and Small (S)-segment.^17^ The S-segment is ambisense; a negative-sense S-segment RNA encodes nucleoprotein (N protein) mRNA and a positive-sense S-segment RNA encodes nonstructural protein (NSs protein) mRNA. The M-segment encodes 78kD, NSm, Gn, and Gc proteins in a single open-reading frame, and these proteins are co-translationally cleaved from precursor proteins.^18, 19^ The L-segment encodes an RNA-dependent RNA polymerase (L protein). The MP-12 strain encodes 23 unique genetic mutations (Fig. 1): four mutations in the untranslated region, ten silent mutations, and nine amino acid substitutions. The MP-12 vaccine is attenuated through a combination of mutations in the L-segment, M-segment, and S-segment.^20^ Mutations Gn-U795C (Y259H) and Gc-A3564G (R1182G) independently contribute to the partial attenuation of MP-12 strain through the M-segment, and a combination of mutations Gn-U795C (Y259H), Gc-A3564G (R1182G), and L-G3104A (R1029K) is sufficient to abolish the virulence of ZH501 strain in mice. Temperature-sensitive (ts) mutations were also identified for the MP-12 strain.^21^ MP-12 replication was restricted at 38 °C and above in Vero and MRC-5 cells. The L-segment, M-segment, and S-segment independently contribute to the ts phenotype of MP-12, and two mutations in the M-segment (Gn-U795C [Y259H] and Gc-A3564G [R1182G]) and two mutations in the L-segment (L-U533C [V172A] and L-G3750A [M1244I]) are independently responsible for the ts phenotype (Fig. 1).

The genetic stability of the MP-12 vaccine has been previously evaluated. Through a high throughput sequencing approach, MP-12 vaccine Lot-7-2-88 has been shown to not contain detectable genetic subpopulations encoding the parental ZH548 strain sequence at its 23 mutation sites, which are unique to the MP-12 strain.^22^ MP-12 isolates from human plasma or buffy coats after vaccination have also been genetically characterized.^15^ Ten mutations were identified in seven isolates, but none of them were reversion mutations to the parental ZH548 strain. To date, there is no evidence of reversion in the MP-12 vaccine lot or human isolates.

Considering the plasticity of the RNA virus genome, due to an error-prone RNA polymerase, the MP-12 vaccine could revert to the parental RVFV sequence during multiple cycles of viral replications. For example, MP-12 RNA synthesis is decreased at 37 °C due to the L-G3750A (M1244I) ts mutation.^21^ If a reversion mutation occurs by chance, the viral genetic subpopulation could be replaced due to the above-mentioned disadvantage in viral RNA synthesis. To address this possibility, the MP-12 vaccine Lot-7-2-88 was serially passaged in MRC-5 cells or Vero cells. Reversion mutations at five selected mutation sites (Gn-U795C [Y259H], Gc-A3564G [R1182G], L-U533C [V172A], L-G3104A [R1029K], and G3750A [M1244I]) were quantitatively analyzed by droplet digital polymerase chain reaction (ddPCR). We also hypothesized that the mutant rMP12-ΔNSs16/198, which lacks functional NSs, is genetically less stable than parental MP-12 in Vero cells. RVFV NSs causes the cessation of cellular general transcription, including the interferon (IFN)-β gene, and promotes posttranslational degradation of dsRNA-dependent protein kinase (PKR) and transcription factor IIH.^23^ Without NSs expression, host selection pressure would be expected to increase due to the presence of endogenous active PKR or active host transcription. We also determined if replication of the MP-12 strain causes reversion at any of those five mutation sites in mice, using viral RNA extracted from brains of mice suffering viral encephalitis after infection with MP-12 or a recombinant MP-12 strain encoding the Toscana virus (TOSV) NSs gene in the place of the MP-12 NSs gene (rMP12-TOSNSs).^24^

## Results

### Reversion mutations in MP-12 and rMP12-ΔNSs16/198 during viral passages in culture cells

**Fig. 2:**
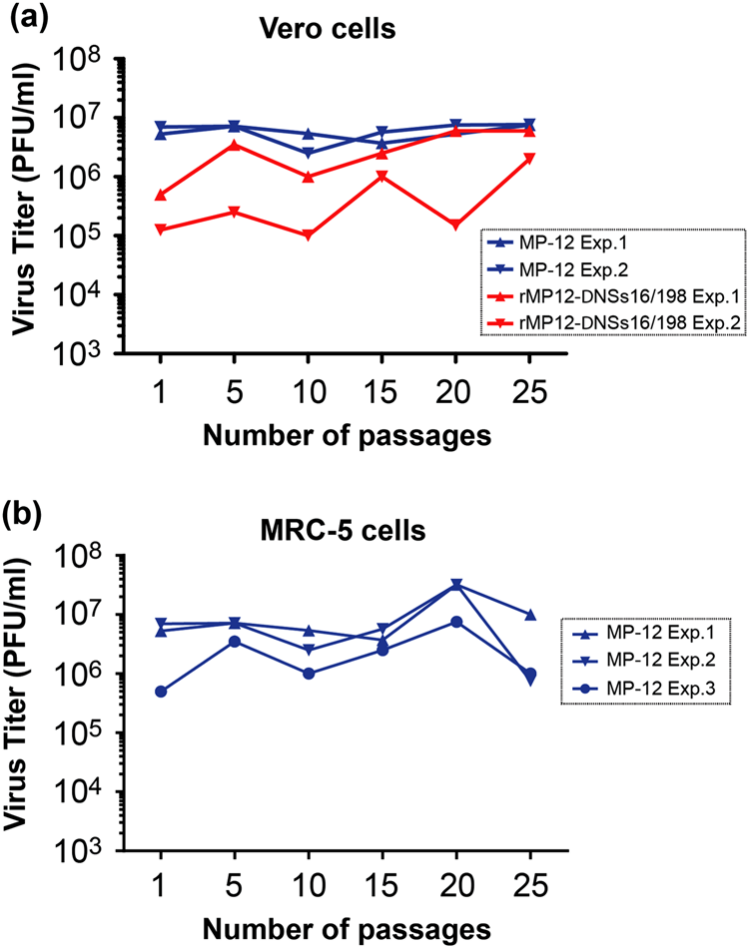
Virus titers during serial virus passages. **a** Vero cells were infected with either MP-12 vaccine Lot-7-2-88 or rMP12-ΔNSs16/198 at MOI of 0.01 (two separate passage experiments under the same conditions: Exp-1 and 2). At 3 dpi, culture supernatants were collected, and 0.1 μl was used for the next passage in Vero cells (5 × 10^5^ cells). Virus titers in culture supernatants at 3 dpi of passages 1, 5, 10, 15, 20, and 25 are shown in a graph. **b** Similarly, MRC-5 cells were infected with MP-12 vaccine Lot-7-2-88 (three separate passage experiments under the same conditions: Exp-1, 2, and 3), and virus titers were measured at passages 1, 5, 10, 15, 20, and 25

The MP-12 vaccine Lot-7-2-88 was serially passaged 25 times in Vero cells. The deletion of the RVFV S-segment is known to occur during serial passages in BHK-21 cells (0.1 MOI),^25^ and high MOI passage of virus may also lead to the generation of defective interfering (DI) particles.^26^ To minimize the generation of RVFV-encoding gene truncation, the MP-12 passage experiment was therefore designed not to exceed an MOI of 0.01 (Fig. 2). Back titration of culture supernatants at 1, 5, 10, 15, 20, and 25 passages estimated the MOI of each passage to be 0.00002 to 0.002. Similarly, the MP-12 vaccine Lot-7-2-88 was also serially passaged 25 times in MRC-5 cells.

**Fig. 3:**
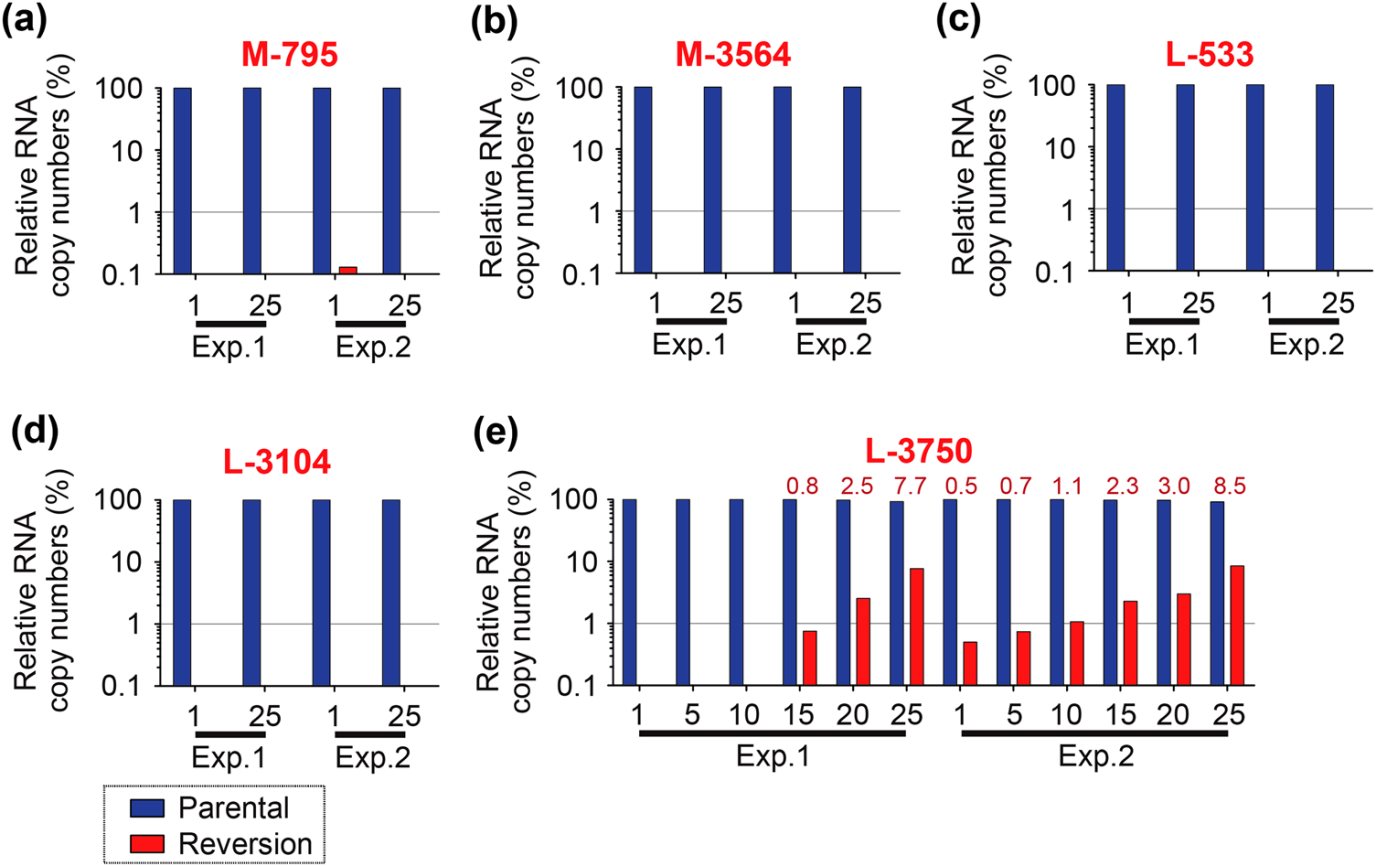
Detection of reversion mutants of the MP-12 vaccine during viral passages in Vero cells. The MP-12 vaccine was passaged 25 times in Vero cells at MOI of 0.01 or less at 3 days post infection. Virus RNA was purified from culture supernatants and analyzed for the presence of reversion mutations using Taqman probes (shown in Supplementary Fig. 1 and Supplementary Table 2). **a** M-segment nt. 795: HEX-795-C and FAM-795-T, **b** M-segment nt. 3564: HEX-3564-G and FAM-3564-A, **c** L-segment nt. 533: HEX-533-C and FAM-533-T, **d** L-segment nt. 3104: HEX-3104-A and FAM-3104-G, and **e** L-segment nt. 3750: HEX-3750-A and FAM-3750-G. Two independent studies are shown (Exp-1 and 2). Relative percentages of RNA copy numbers for parental and reversion mutants are shown in *blue* and *red bars*, respectively. The bar labels in *red* represent the percentages of reversion mutants

**Fig. 4:**
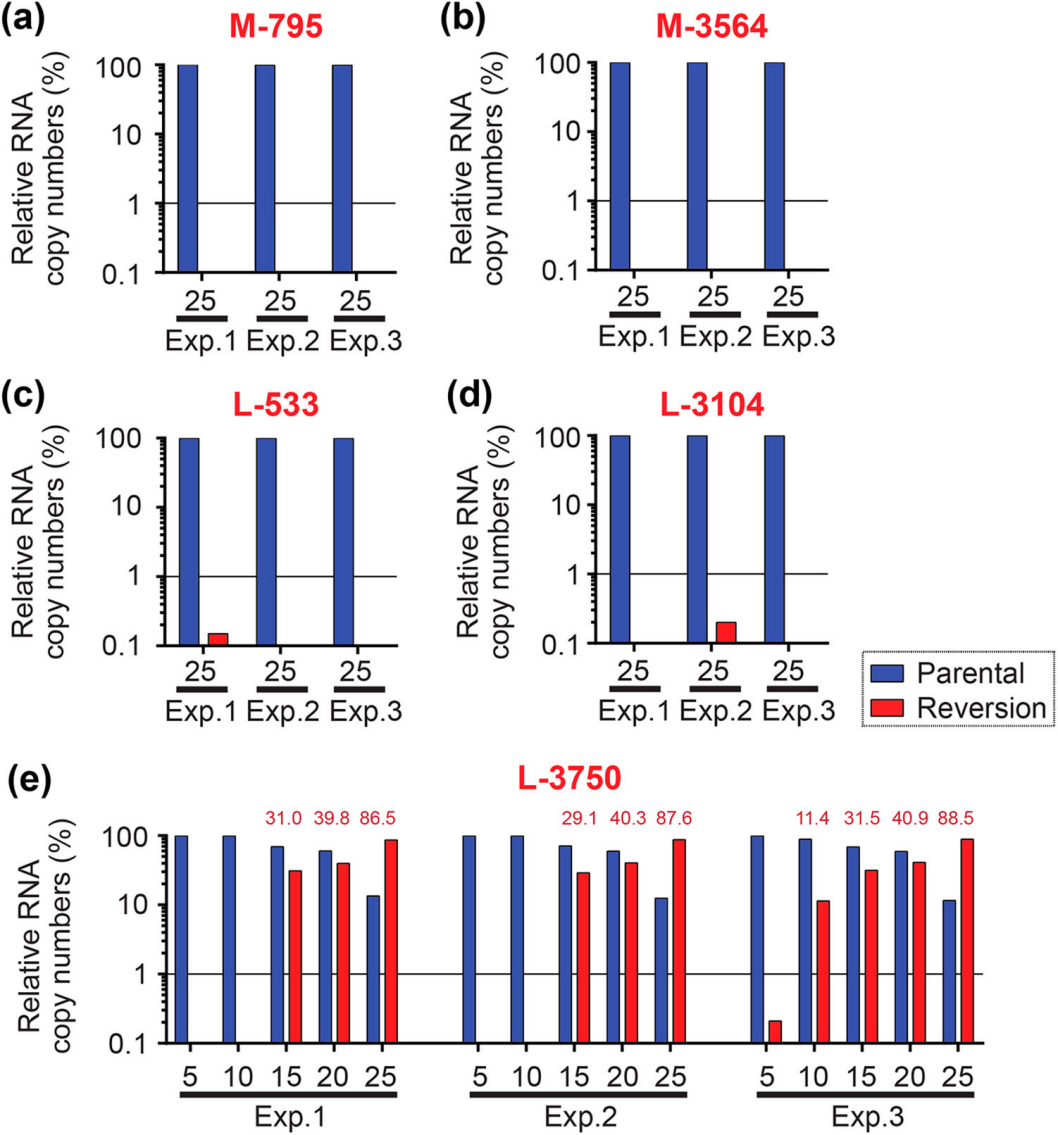
Detection of reversion mutants of the MP-12 vaccine during viral passages in MRC-5 cells. The MP-12 vaccine was passaged 25 times in MRC-5 cells at MOI of 0.01 or less at 3 days post infection. Virus RNA was purified from culture supernatants and analyzed for the presence of reversion mutations using Taqman probes (shown in Supplementary Fig. 1 and Supplementary Table 2). **a** M-segment nt. 795: HEX-795-C and FAM-795-T, **b** M-segment nt. 3564: HEX-3564-G and FAM-3564-A, **c** L-segment nt. 533: HEX-533-C and FAM-533-T, **d** L-segment nt. 3104: HEX-3104-A and FAM-3104-G, and **e** L-segment nt. 3750: HEX-3750-A and FAM-3750-G. Three independent studies are shown (Exp-1, 2, and 3). Relative percentages of RNA copy numbers for parental and reversion mutants are shown in *blue* and *red bars*, respectively. The bar labels in *red* represent the percentages of reversion mutants

Five MP-12 mutations—M-795, M-3564, L-533, L-3104, and L-3750—were analyzed for reversion due to their critical contribution to the attenuated or ts phenotypes.^20, 21^ The ddPCR analyses demonstrated that only one of the analyzed mutations showed reversion mutations exceeding 0.2% of the population during passaging (Fig. 3). As shown by the bars labeled in *red* in Fig. 3e, a genetic subpopulation encoding a reversion at L-3750 emerged at passage 15 in Exp-1 (0.8%) and at passage 1 in Exp-2 (0.5%), and gradually increased to 8% by passage 25 (Exp-1 and 2). In MRC-5 cells, the MP-12 vaccine also demonstrated no increases in genetic subpopulations encoding reversions in M-795, M-3564, L-533, or L-3104 exceeding 0.2% (Fig. 4); however, as shown by the bars labeled in *red* in Fig. 4e, a reversion at L-3750 occurred at passage 15 (31% of population in Exp-1; 29%, Exp-2) or passage 10 (11%, Exp-3), and the reversion population became dominant at passage 25 (87–89%). Taken together, these results demonstrated that L-3750 can be reverted during passages in Vero or MRC-5 cells, and the domination of the L-3750 reversion mutant occurs faster in MRC-5 cells than in Vero cells.

**Fig. 5:**
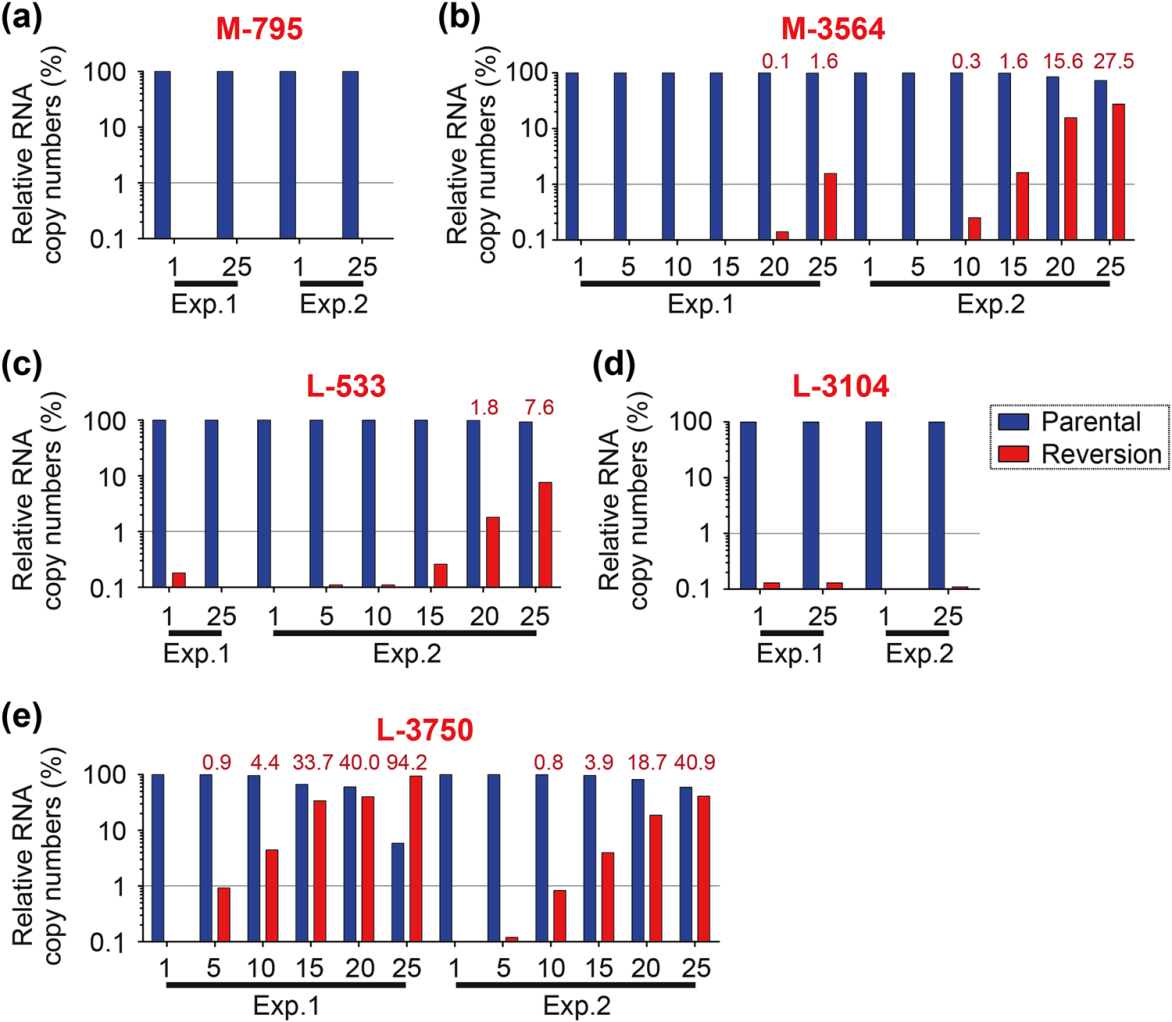
Detection of reversion mutants of rMP12-ΔNSs16/198 during passages in Vero cells. Recombinant MP-12 encoding a 69% in-frame truncation of NSs open reading frame (rMP12-ΔNSs16/198) was passaged 25 times in Vero cells at MOI of 0.01 or less at 3 days post infection. The presence of reversion mutations are shown. **a** M-segment nt. 795: HEX-795-C and FAM-795-T, **b** M-segment nt. 3564: HEX-3564-G and FAM-3564-A, **c** L-segment nt. 533: HEX-533-C and FAM-533-T, **d** L-segment nt. 3104: HEX-3104-A and FAM-3104-G, and **e** L-segment nt. 3750: HEX-3750-A and FAM-3750-G. Two independent studies are shown (Exp-1 and 2). Relative percentages of RNA copy numbers for parental and reversion mutants are shown in *blue* and *red* bars, respectively. The bar labels in *red* represent the percentages of reversion mutants

RVFV lacking a functional NSs can replicate efficiently in type-I IFN-incompetent cells, such as Vero or BHK-21 cells,^27–30^ but less efficiently in type-I IFN-competent cells, such as MRC-5 cells.^29, 30^ In this study, RVFV lacking a functional NSs (rMP12-ΔNSs16/198) was passaged 25 times in Vero cells to investigate its genetic stability (Fig. 2). The ddPCR analysis revealed no reversion mutations at M-795 or L-3104 (Fig. 5a, d); however, as shown by the bars labeled in *red* in Fig 5b, c, e, reversions at M-3564 (2% of population, Exp-1 p25; 28%, Exp-2 p25), L-533 (8%, Exp-2 p25), and L-3750 (94%, Exp-1 p25; 41%, Exp-2 p25) were detected during 25 passages. To check the accuracy of ddPCR, the reversions of rMP12-ΔNSs16/198 (Exp-1) at M-3564 and L-3750 were further validated by MiSeq deep sequencing using PCR fragments (Exp-1, passage 25 sample). This analysis demonstrated the reversion of M-3564 in 5.7% of the population and L-3750 in 82.7%, which was consistent with our ddPCR results (Table 1). Conversely, variants encoding reversion mutations were not detectable at M-795 and L-3104. Thus, our results confirmed that replication of rMP12-ΔNSs16/198 leads to the reversion mutation at M-3564, L-533, and L-3750.

**Table 1:** Illumina MiSeq amplicon sequencing for rMP12-ΔNSs16/198 (Vero cells, Exp-1, p25)

Segment	PCR fragment^a^	Location	nt. mutation	aa. mutation	Variant reads	Total coverage	%
M	nt.705-1165	719	U to A	S to R	28992	267685	10.8
		877	A to U	Q to L	202505	601576	33.7
		1163	C to U	–	159382	910283	17.5
		1165	A to G	K to R	344469	866107	39.8
	nt.3369-3815	3412	C to U	T to I	10400	863570	1.2
		3555	U to C	Y to H	39842	2246190	1.8
		3564^b^	G to A	G to R	124721	2182917	5.7
		3684	C to U	–	96600	2129301	4.5
		3733	A to G	–	39842	2246190	1.3
L	nt.2850-3295	3240	C to A	–	47726	2514812	1.9
	nt.3733-4170	3750^b^	A to G	I to M	438643	530118	82.7
		3814	U to C	–	944868	995347	94.9
		3816	A to C	–	944042	995263	94.9
		4142	A to C	I to T	5485	416681	1.3

The PCR fragments were amplified from viral RNA extracted from culture supernatants of rMP12-ΔNSs16/198 (Exp-1) at passage 25. The threshold of variant detection was set as 1%. nt., nucleotide; aa., amino acid

^a^ Nucleotide positions of PCR fragments in the antiviral-sense M- or L-segment. Primer regions are excluded

^b^ Reversion mutations to parental ZH548 strain

In order to analyze overall mutations of passaged viruses, plaque clones of MP-12 (Vero cell passage 25, Exp-1) and rMP12-ΔNSs16/198 (Vero cell passage 25, Exp-1) were isolated, and the full-length genome sequence of each of four plaques was determined. Differences between these sequences and the original strains were then identified (Supplementary Table 1). All four clones of rMP12-ΔNSs16/198 encoded a reversion mutation at L-3750, and an additional 14 mutations were identified in the L-segment, M-segment, and S-segment. Among them, the M-A877U (three of four clones) and M-A1165G (two of four clones) mutations were also identified by deep sequencing, as 33.7 and 39.8% of the population, respectively (Table 1). MP-12 plaque clones did not encode reversion mutations, yet 18 mutations were identified among four clones. None of the mutations found in MP-12 or rMP12-ΔNSs16/198 were identical to those previously found in MP-12 plaque isolates from human vaccinees.^15^

### Analysis of reversion mutations in vivo in a mouse encephalitis model

**Fig. 6:**
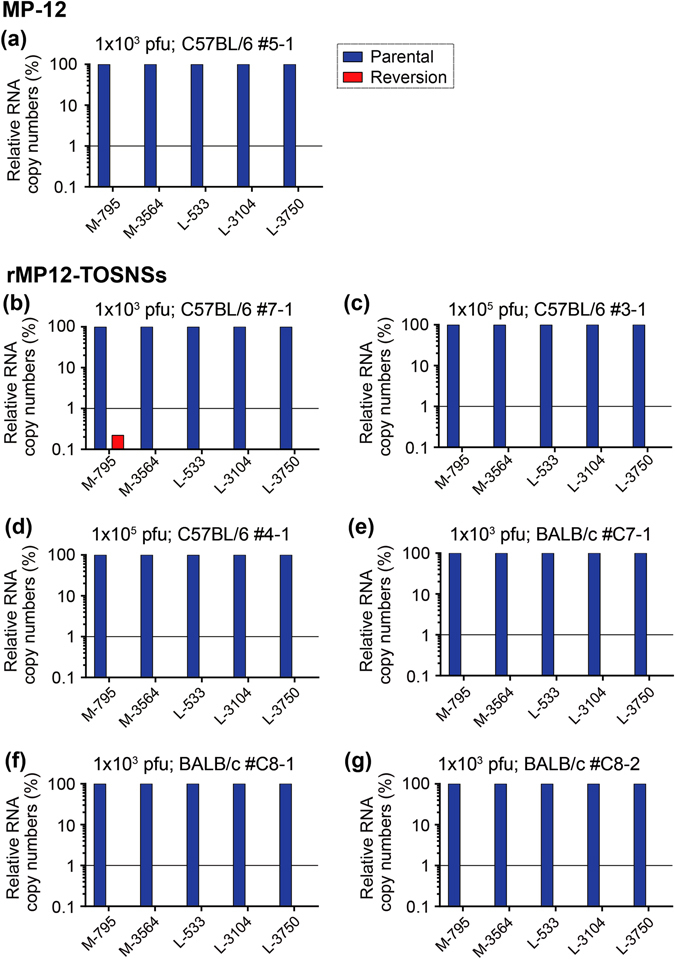
Detection of reversion mutations in MP-12 and rMP12-TOSNSs in brains of mice. The ddPCR to detect reversion mutants at M-795, M-3564, L-533, L-3104, and L-3750 was performed using total RNA samples from mouse brains reported by Indran et al.^24^ BALB/c (**e**–**g**) or C57BL/6 mice (**a**–**d**) were inoculated with either MP-12 (**a**) or rMP12-TOSNSs (**b**–**g**) at either 1 × 10^3^ or 1 × 10^5^ pfu (intraperitoneally). Relative percentages of RNA copy numbers for parental and reversion mutants are shown in *blue* and *red bars*, respectively

Although our study indicated that L-3750 has a risk of reversion during extensive viral passages in cell culture, reversions of MP-12 mutations have not been found in MP-12 isolates from vaccinees.^15^ We have previously shown that subcutaneous vaccination of mice with the MP-12 vaccine rarely causes viral encephalitis,^31, 32^ whereas MP-12 encoding TOSV NSs in place of MP-12 NSs (rMP12-TOSNSs) causes viral encephalitis in up to 40% of mice at 10 to 18 dpi.^24^ To evaluate the occurrence of reversion mutations of MP-12 and rMP12-TOSNSs during viral replication in vivo, total RNAs were extracted from the brains of mice with viral encephalitis caused by MP-12 (*n* = 1) or rMP12-TOSNSs (*n* = 6),^24^ and ddPCR analysis was conducted to detect reversion mutations. None of the seven brain samples, however, showed reversion mutations at M-795, M-3564, L-533, L-3104, or L-3750 (Fig. 6). Our results indicate that the replication of MP-12 or rMP12-TOSNSs in vivo for more than 10 days does not allow the emergence of reversion mutants.

## Discussion

Live-attenuated vaccines have played historical roles in dramatically decreasing human and animal diseases of major public health importance.^33–35^ In contrast to the benefit of strong immunogenicity, it is often difficult to explain the mechanism of attenuation due to empirical attenuation approaches.^36^ As a result, reversion to wild-type virulence has been a concern for massive vaccination at the cost of disease control.^37^ Reversion occurs through in several ways, including recombination between vaccine strains and pathogenic strains,^38–41^ back-mutations of attenuation mutations,^42, 43^ or compensation mutations^44^ in the genome. The homologous recombination of negative-strand RNA viruses is relatively rare,^45^ whereas attenuated viral phenotypes can be altered during replication. Re-adaptation of the cold-adapted 2009 pandemic H1N1 live-attenuated FluMist vaccine at 37 °C resulted in reversion of a ts phenotype and virulence by acquisition of compensation mutations, without back-mutations of responsive ts mutations.^46^

RVFV is a three-segmented negative-stranded RNA virus belonging to the family *Bunyaviridae*. Few veterinary vaccines (e.g., Smithburn and Clone 13 vaccines) have been licensed for the use in Africa.^47, 48^ RVFV, including the above-mentioned vaccine strains, is classified as a select agent in the US, whereas a live-attenuated MP-12 strain is exempted from the select agent rules.^2, 49^ The safety and immunogenicity of the MP-12 vaccine has been demonstrated in sheep and cattle, and thus, this vaccine is conditionally licensed for veterinary use in the US.^16, 50–56^ The MP-12 vaccine has also been evaluated in human volunteers for the safety and immunogenicity.^14, 15^ The MP-12 vaccine Lot 7-2-88 does not contain any detectable viral subpopulations encoding reversion mutations.^22^ Furthermore, vaccination using the MP-12 vaccine lot did not cause detectable reversion mutation in human vaccinees.^15^ Thus, reversion to virulence appears not to be a major concern for the MP-12 vaccine as long as it is produced from an appropriate Seed Lot system. Although reversion mutants have never been an issue for MP-12 vaccine, thus far, no previous study has accurately demonstrated changes in viral genetic subpopulations of the MP-12 vaccine strain during viral replications. With an increased population of reversion mutants in viral subpopulations, a vaccine may have an increased risk of adverse effects in vaccinees.

Attenuation of the MP-12 vaccine occurs due to a combination of several mutations in the L-segment, M-segment, and S-segment, and the mutations in the L-segment and M-segment play a major role in attenuation of MP-12.^20, 57^ Although a combination of each mutation is important, some mutations independently show attenuation and/or ts phenotype, including M-U795C (Y259H), M-A3564G (R1182G), L-U533C (V172A), and L-G3750A (M1244I).^20, 21^ A previous study showed that the RVFV ZH548 strain causes a truncation of the NSs gene during serial passages in either BHK-21 cells or Aag2 cells, even at a MOI of 0.1.^25^ To prevent the emergence of gene truncation or DI RNA formation, serial passage was performed at very low MOI in the current study. The back titration of samples measured the MOI range as being between 0.00002 and 0.002 (10^1^ to 10^4^ viruses per input) during the passage. Under these experimental conditions, the sampling of limited amounts of viruses at each passage randomly selected specific viral populations. It is unlikely that mutants would become a dominant population unless the parental virus is not adapted to the cell line.^58^ This study clearly demonstrated that the MP-12 vaccine increases viral populations encoding the back-mutation L-A3750G (I1244M) during serial viral passages in Vero or MRC-5 cells, which is a reversion mutation to ZH548. Emergence of the I1244M mutation in two or three independent experiments indicates that this mutation has an advantage in viral replication over the parental genotype. In fact, the L-M1244I mutation is responsible for the ts phenotype of the L-segment, and decreases viral RNA synthesis, but not viral titer, at 37 °C.^21^ Thus, it is plausible that a temperature of 37 °C was a limiting factor for the RNA replication of the parental genotype, and thus the selection of the reversion mutant at L-M1244I occurred.

Reversion mutants can occur through the introduction of mutation by an error-prone RNA-dependent RNA polymerase, followed by subsequent selection of genotype during viral replication. The rMP12-ΔNSs16/198 was generated from cloned cDNA using reverse genetics. A possible source of reversion mutation was therefore the error-prone RVFV L protein. The mutation rates of negative-strand RNA viruses range from 10^−4^ to 10^−6^ per nucleotide per cell infection.^36, 59^ Since the total length of the MP-12 genome is 11,979 nt, only 0.01–1 mutations are expected to be introduced per a single-step virus life cycle. At a very low MOI, input virus is required to repeatedly infect neighboring cells, which can increase the possibility of the introduction of new mutations. On the other hand, the serial passages may not have allowed subsequent selection of specific reversion mutants in culture cells, unless the mutations can change viral phenotype.

RVFV NSs protein is a major virulence factor for RVF, and inhibits host antiviral responses via various mechanisms, including the cessation of cellular transcription and the degradation of PKR.^60–66^ Thus, pathogenic RVFV strains have been attenuated through the deletion of NSs gene for the development of live-attenuated RVF vaccines.^29, 67–69^ The rMP12-ΔNSs16/198 strain (formerly named rMP12-C13type) encodes an in-frame 69% truncation in the NSs gene in the MP-12 strain backbone, causing viral replication to be limited in type-I IFN competent cells.^30^ Type-I IFN-deficient Vero cells are an animal cell line established for vaccine production, and are also suitable for the growth of the rMP12-ΔNSs16/198 strain.^27, 70, 71^ We investigated whether or not a lack of the NSs gene affects the selection of a viral genetic subpopulation during replication in Vero cells. With a lack of functional NSs, the rMP12-ΔNSs16/198 demonstrated emergence of reversion mutants not only at M1244I, but also at M-A3564G and L-U533C. A lack of NSs protein expression could therefore result in a change in selection threshold for reversion mutants, due to the cellular antiviral activities, which are not inhibited by NSs proteins; e.g., active cellular transcription and the expression of endogenous PKR.

Reversion mutations were further analyzed using an in vivo model. A recombinant MP-12 strain encoding the TOSV NSs gene (rMP12-TOSNSs) causes viral encephalitis in up to 40% of infected mice, whereas the neuroinvasion of parental MP-12 strain is less frequent.^24^ Robust replication of MP-12 and rMP12-TOSNSs occurred in the brain at 10 dpi or later; although the mechanism of neuroinvasion has not been elucidated, it was initially assumed that pathogenic reversion mutants of the MP-12 strain selectively invade the central nervous system in mice. The expression of TOSV NSs proteins in the place of MP-12 NSs proteins may also affect the threshold of the mutant selection process. Contrary to the hypothesis, viral RNA from the brains of mice did not contain detectable reversion mutations, indicating that neuroinvasion by the MP-12 or rMP12-TOSNSs strain is an intrinsic to the nature of the parental genotype, and is independent of reversion mutations in the L-segment and M-segment. It should be noted that susceptibility to RVFV differs largely among host species and ages; the neuroinvasion of MP-12 strain is not known to occur in humans or ruminants.^7, 72^

An individual reversion mutation does not immediately generate a pathogenic mutant of the MP-12 strain, because residual mutations alternatively attenuate the virus.^20^ For example, the MP-12 strain encoding reversion mutations of both M-H259Y and M-G1182R in the M-segment was still largely attenuated in mice. Two of MP-12 L-segment mutations (L-V172A and L-M1244I) are ts mutations, and each inhibits viral RNA synthesis in a temperature-dependent manner.^21^ The threshold of restriction temperature for MP-12 virus replication was 38 °C. The attenuation of L-segment mutations, however, cannot be accurately evaluated using a mouse model, because none of recombinant ZH501 strains, encoding one of the MP-12 L-segment mutations were attenuated in mice.^20^ Although the normal temperature of mice appears not to restrict the replication of the MP-12 strain, normal body temperatures of ruminants are higher than those of mice.^73, 74^ It is therefore possible that the attenuation of MP-12 strain is supported by ts mutations in ruminants, but not in mice. Previous studies have shown that the MP-12 strain has residual virulence in ewes, cows, and alpacas.^50, 75–77^ Virological and genetic analyses of viral isolates (e.g., compensation mutations) from affected tissues in ruminants may aid in elucidating the mechanism of residual virulence in the MP-12 strain.

The genetic stability of live-attenuated vaccine should be carefully analyzed if massive vaccination will be conducted in livestock animals or humans. Overall, our study showed that the MP-12 vaccine has outstanding genetic stability in Vero and MRC-5 cells within limited viral replication cycles. Nonetheless, reversion mutations at L-V172A, L-M1244I, and M-R1182G were confirmed during serial passage of viruses. Thus, in further vaccine safety analyses of the MP-12 vaccine strain, attention should be paid to the virulence associated with mutant genotypes generated in vitro and in vivo.

## Methods

### Media, cells, and viruses

Viruses were passaged in Vero cells (ATCC CCL-81) and MRC-5 cells (ATCC CCL-171); VeroE6 cells (ATCC CRL-1586) were used for virus titration. Cells were maintained in Dulbecco’s modified minimum essential medium containing 10% fetal bovine serum (FBS), penicillin (100 U/ml), and streptomycin (100 μg/ml). To rescue recombinant MP-12, BHK/T7–9 cells stably expressing T7 RNA polymerase were used^30, 78^; these cells were maintained in MEM-alpha containing 10% FBS, penicillin/streptomycin, and 600 µg/ml of hygromycin B. All cells used in this study were verified to be mycoplasma free (UTMB Tissue Culture Core Facility), and the identity of MRC-5 cells was authenticated by short tandem repeat analysis (UTMB Molecular Genomics Core Facility). The MP-12 vaccine Lot 7-2-88 was obtained from Dr. John C. Morrill at the University of Texas Medical Branch at Galveston (UTMB), and used for virus serial passage experiments. Recombinant MP-12 (rMP-12) encoding an in-frame 69% truncation of NSs similar to that of the Clone 13 strain (rMP12-ΔNSs16/198, other name: rMP12-C13type)^29^ has been previously described.^30^ The rMP-12 strain encoding TOSV NSs in the place of MP-12 NSs (rMP12-TOSNSs) has been described previously.^24, 79^ Rescued viruses were amplified once in VeroE6 cells after recovery in BHK/T7-9 cells, titrated by plaque assay, and then used for subsequent experiments.

### Serial passages of MP-12 and rMP12-ΔNSs16/198

Vero cells were infected with MP-12 vaccine Lot 7-2-88 or rMP12-ΔNSs16/198 at 0.01 MOI (two separate passage experiments under the same conditions: Exp-1 and Exp-2). Culture supernatants were then collected at 3 days post infection (dpi), and 0.1 μl of supernatant was blindly passaged to fresh Vero cells in a 12-well plate (5 × 10^5^ cells per well). When passage 25 was achieved, culture supernatants were back-titrated at 1, 5, 10, 15, 20, and 25 passages, and the MOI of each passage was determined to be 0.00002–0.002 (Fig. 2). The same passage experiment was repeated in MRC-5 cells using MP-12 vaccine Lot 7-2-88 (three separate passage experiments under the same conditions: Exp-1, Exp-2, and Exp-3) (Fig. 2). The rMP12-ΔNSs16/198 was not tested in MRC-5 cells, because this virus lacks the expression of functional NSs proteins and does not efficiently replicate in type-I IFN-competent MRC-5 cells.^30^

### ddPCR analysis

To measure the RNA copy numbers of parental MP-12 and the mutant genotypes in viral M-segment and L-segment RNA, ddPCR was performed using the BioRad QX100 droplet generator and reader, as described previously.^80^ Two Taqman probes were designed per mutation (Supplementary Fig. 1). The specificity and sensitivity of detecting mutant genotypes was evaluated in each probe set using in vitro synthesized M-segment or L-segment RNA from parental and mutant genotypes (Supplementary Fig. 2). To extract viral RNA, 250 μl of clarified culture supernatants were digested with 25 U Benzonase (EMD Millipore) at 37 °C for 30 min, to minimize the contamination of free viral RNA derived from dead cells. The enzyme-treated culture supernatant (250 μl) was mixed with 750 μl Trizol LS (Life Technologies), and spike RNA (in vitro synthesized chloramphenicol acetyltransferase RNA, 3 μg) was added. The spike RNA was included because the quantity of viral RNA in a 250 μl sample was too low to be measured accurately. Viral RNA was then extracted using Direct-zol RNA MiniPrep Kit (Zymo Research), according to manufacturer’s instructions. The concentration of extracted RNA (spike RNA) was measured via Qubit Fluorometer (Life Technologies) and first-strand complementary DNA (cDNA) was synthesized using iScript (BioRad), which has RNase H activity that can digest template RNA upon cDNA synthesis and therefore better reflects the ratio of cDNA copy number per RNA template. PCR reactions were prepared as follows: 250 nM of each Taqman probe, 900 nM of each primer, ddPCR Supermix for Probes (BioRad), cDNA, and water (up to 25 μl). PCR cycling parameters were: initial denaturation (95 °C for 10 min), followed by 40 cycles of 94 °C for 30 s, 60 °C for 1 min, and a final denaturation step of 98 °C for 10 min. The number of droplets with positive and negative signals was measured using a BioRad QX100 droplet reader. Finally, data analysis was performed using QuantaSoft Version 1.4 (BioRad). The droplet reader was able to count up to 20,000 droplets and the input of cDNA was optimized to visualize two genotype populations by ddPCR.

### Deep sequencing of PCR fragments and variant analysis

To validate the accuracy of the ddPCR results, deep sequencing using an Illumina MiSeq Desktop Sequencer was performed (Applied Biological Materials Inc). For this purpose, the 25^th^ passage sample of rMP12-ΔNSs16/198 (Exp-1) was used for analysis of four sites. Two sites possessed reversion mutations that were detectable by ddPCR (M-A3564G or L-G3750A), and the other two sites possessed reversion mutations that were not detectable by ddPCR (M-U795C and L-G3104A). One ng of PCR fragments flanking either M-U795C (nt. 705–1165), M-A3564G (nt. 3360–3815), L-G3104A (nt. 2850–3295), or L-G3750A (nt. 3733–4170) were fragmented with the Nextera XT DNA Library Preparation Kit, and sequenced in a paired-end run of 250 base pairs using the MiSeq Reagent Kit v2 (500 cycles). Resulting FASTAQ files were imported to the CLC Genomics Workbench 7.0.4. as paired reads, and failed files were excluded. Probabilistic variant detection was performed to detect 1% minimum variant frequency found in both pairs, with a minimum central quality of 20 and a minimum neighborhood quality of 15, within five nucleotides.

### Analysis of viral RNA from brains of mice

For the analysis of genetic changes in the MP-12 strain in vivo, total RNAs were extracted from brain tissue samples stored in TRIzol, which were collected from 4-week-old female BALB/c mice or C57BL/6 mice intraperitoneally vaccinated with MP-12 (*n* = 10 each) or rMP12-TOSNSs (*n* = 10 each) (1 × 10^3^ pfu or 1 × 10^5^ pfu) in a previous experiment.^24^ In that previous experiment, encephalitis was confirmed by following mice during a 56-day observation period. During that time, encephalitis was found in 10% of mice vaccinated with 1 × 10^3^ pfu of MP-12, at 11 dpi in BALB/c mice (sample not available) and at 14 dpi in C57BL/6 mice (mouse #5-1). No mice, regardless of strain, developed encephalitis when vaccinated with 1 × 10^5^ pfu of MP-12. When vaccinated with 1 × 10^3^ pfu of rMP12-TOSNSs, 30% of BALB/c mice (11 dpi: mouse #C7-1, 11 dpi: mouse #C8-1, 15 dpi: mouse #C8-2) and 10% of C57BL/6 mice (12 dpi: mouse #7-1) developed encephalitis. Finally, vaccination with 1 × 10^5^ pfu of rMP12-TOSNSs caused no encephalitis in BALB/c mice, but 30% of C57BL/6 mice developed encephalitis (10 dpi: mouse #3-1, 12 dpi: mouse #4-1).^24^

### Ethics statement

All experiments using recombinant RVFV were performed with the approval of the Notification of Use by the Institutional Biosafety Committee at UTMB. Mouse experiments were performed in facilities accredited by the Association for Assessment and Accreditation of Laboratory Animal Care, in accordance with the Animal Welfare Act, NIH guidelines, and US federal law. Animal protocols were approved by the UTMB Institutional Animal Care and Use Committee, Protocol number 0904027.

### Data availability

The raw sequencing data by MiSeq was deposited in the Sequence Read Archive database (accession number: SRP103530). The S-segment sequence of rMP12-ΔNSs16/198 was deposited in the Genbank (accession number: KY926697). The M-segment and L-segment sequences of rMP12-ΔNSs16/198 were identical to that of MP-12 strain (Genbank accession numbers: DQ380208 and DQ375404).^5^

## Electronic supplementary material

Supplementary Figure 1

Supplementary Figure 2

Supplementary Table 1

Supplementary Table 2
