# Supplementary material for: Genetic stability of Rift Valley fever virus MP-12 vaccine during serial passages in culture cells
Source: NPJ Vaccines. 2017 Jul 17;2:20. doi: 10.1038/s41541-017-0021-9 (PMC5627234; doi:10.1038/s41541-017-0021-9)
Supplement: Supplementary file 1 — Supplementary Figure 1 [file 41541_2017_21_MOESM1_ESM.pdf]

Supplementary Figure 1

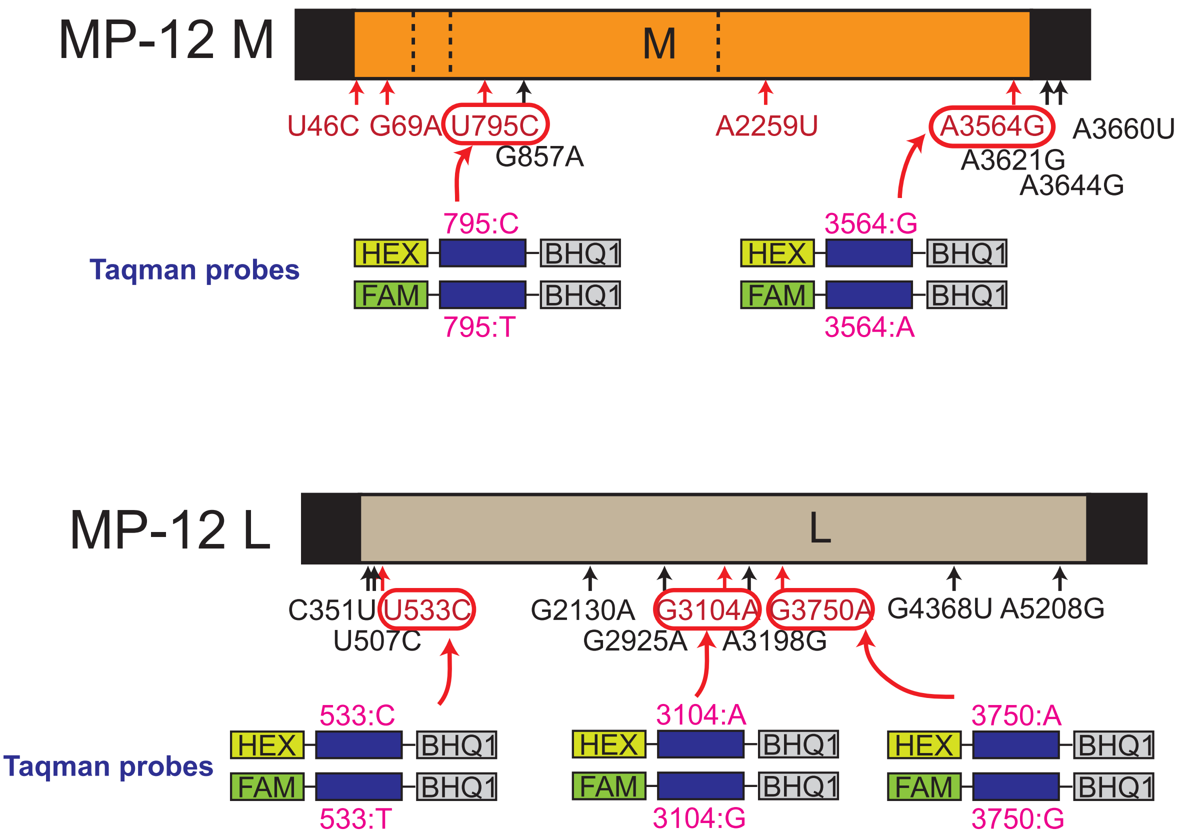

Supplementary Figure 1. Probe design for M-795, M-3564, L-533, L-3104, and L-3750. We designed two Taqman DNA probes at the M-795, M-3564, L-533, L-3104, and L-3750 sites. One probe was 5' conjugated with either hexachlorofluorescein (HEX; Absorbance max: 535 nm; Emission max: 556 nm) to detect parental MP-12 genotype, or 6-carboxyfluorescein (6-FAM; Absorbance max: 494 nm; Emission max: 518 nm) to detect mutant genotype reverted to pathogenic genotype. The other probe was 3' conjugated with Black Hole Quencher-1 (BHQ-1). Sequences of probes and primers are shown in Supplementary Table 2.
