# Supplementary material for: Genetic stability of Rift Valley fever virus MP-12 vaccine during serial passages in culture cells
Source: NPJ Vaccines. 2017 Jul 17;2:20. doi: 10.1038/s41541-017-0021-9 (PMC5627234; doi:10.1038/s41541-017-0021-9)
Supplement: Supplementary file 2 — Supplementary Figure 2 [file 41541_2017_21_MOESM2_ESM.pdf]

Supplementary Figure 2

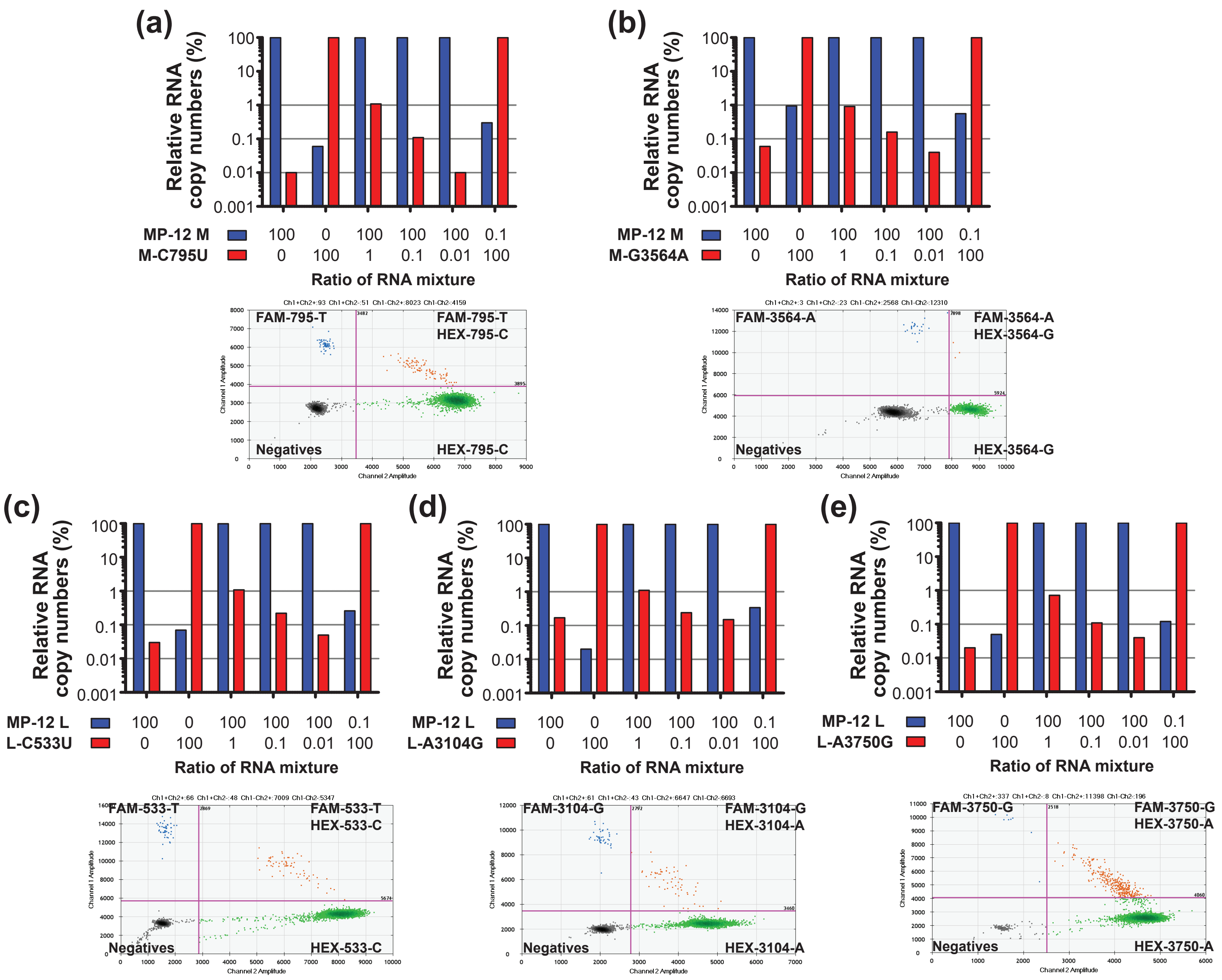

Supplementary Figure 2. Validation of ddPCR using Taqman probes for M-795, M-3564, L-533, L-3104, and L-3750. The accuracy of ddPCR assays were validated using five different Taqman probe sets. Full-length MP-12 M- or L-segment RNAs were in vitro synthesized by MEGAscript T7 Transcription kit (Thermofisher), using linearized pProT7-vM(+) or pProT7-vL(+) plasmids, respectively. In vitro synthesized full-length M- or L-segment RNAs encoding M-C795U, M-G3564A, L-C533U, L-A3104G, or L-A3750G (mutant RNA) were prepared. Synthesized parental MP-12 RNA and mutant RNA were mixed at ratios of 100:0, 0:100, 100:1, 100:0.1, 100:0.01, or 0.1:100. First-stranded cDNA was then synthesized and ddPCR was performed, as described in Methods section. The ddPCR results for M-795 (a), M-3564 (b), L-533 (c), L-3104 (d), and L-3750 (e) are shown. The graph represents the relative percentage of parental and mutant genotype RNA copy numbers. Bottom panels represent the raw images of QX100 Droplet Reader output [parental (HEX): mutant (FAM)
