# Supplementary material for: Genetic stability of Rift Valley fever virus MP-12 vaccine during serial passages in culture cells
Source: NPJ Vaccines. 2017 Jul 17;2:20. doi: 10.1038/s41541-017-0021-9 (PMC5627234; doi:10.1038/s41541-017-0021-9)
Supplement: Supplementary file 3 — Supplementary Table 1 [file 41541_2017_21_MOESM3_ESM.docx]

**Supplementary Table 1. Genome sequences of plaque clones of passaged viral samples**

|  | |  | |  | |  | | nt. | aa. | Plaque |
| --- | --- | --- | --- | --- | --- | --- | --- | --- | --- | --- |
| Virus | Segment | | Gene | | Location | | Mutation^1^ | | Mutation^2^ | clone # |
| MP-12 | S | | N | | 183 | | G to A | | D to N | 2,3 |
| Vero P25 Exp-1 |  | | N | | 761 | | C to U | | - | 1,2,3,4 |
|  |  | | NSs | | 908 | | A to G | | M to T | 1,2,3,4 |
|  |  | | NSs | | 1244 | | C to U | | R to K | 3 |
|  |  | | NSs | | 1409 | | G to A | | A to V | 2,4 |
|  |  | |  | |  | |  | |  |  |
|  | M | | 78kD/NSm | | 171 | | G to A | | E to K | 1,2,3,4 |
|  |  | | 78kD/NSm | | 385 | | A to G | | D to G | 1,3 |
|  |  | | Gn | | 587 | | C to U | | - | 1,3,4 |
|  |  | | Gn | | 829 | | A to G | | K to R | 2 |
|  |  | | Gn | | 1149 | | C to U | | H to Y | 2,3,4 |
|  |  | | Gn | | 1204 | | A to G | | K to R | 2,3,4 |
|  |  | | Gn | | 1876 | | A to G | | N to S | 2,3,4 |
|  |  | | Gc | | 2646 | | G to A | | G to R | 1 |
|  |  | | 5’ UTR | | 3648 | | G to A | | - | 3 |
|  |  | |  | |  | |  | |  |  |
|  | L | | L | | 876 | | U to C | | - | 1 |
|  |  | | L | | 2553 | | G to U | | - | 1,2 |
|  |  | | L | | 5005 | | U to C | | Y to H | 1,2,3,4 |
|  |  | | L | | 6066 | | U to C | | - | 1,2,3,4 |
|  |  | |  | |  | |  | |  |  |
| rMP12-ΔNSs16/198 | S | | NSs | | 891 | | U to A | | S to C | 3 |
| Vero P25 Exp-1 |  | |  | |  | |  | |  |  |
|  | M | | 78kD | | 99 | | G to A | | E to K | 2 |
|  |  | | 78kD/NSm | | 361 | | U to A | | I to K | 3 |
|  |  | | Gn | | 877 | | A to U | | Q to L | 2,3,4 |
|  |  | | Gn | | 1165 | | A to G | | K to R | 1,3 |
|  |  | | Gn | | 1852 | | A to G | | K to G | 1,3 |
|  |  | | Gn | | 1861 | | C to A | | P to Q | 2,4 |
|  |  | | Gc | | 2907 | | U to C | | - | 1,2,3,4 |
|  |  | | Gc | | 2909 | | G to C | | L to F | 1,2,3,4 |
|  |  | |  | |  | |  | |  |  |
|  | L | | L | | 231 | | G to A | | - | 3 |
|  |  | | L | | 3022 | | A to G | | T to A | 4 |
|  |  | | L | | 3750^3^ | | A to G | | I to M | 1,2,3,4 |
|  |  | | L | | 4584 | | G to A | | - | 2 |
|  |  | | L | | 4602 | | G to A | | - | 2 |
|  |  | | L | | 4971 | | U to C | | - | 1 |

^1^nt., nucleotide; ^2^aa., amino acid. ^3^Reversion mutation to parental ZH548 strain.
