# Supplementary material for: Genetic stability of Rift Valley fever virus MP-12 vaccine during serial passages in culture cells
Source: NPJ Vaccines. 2017 Jul 17;2:20. doi: 10.1038/s41541-017-0021-9 (PMC5627234; doi:10.1038/s41541-017-0021-9)
Supplement: Supplementary file 4 — Supplementary Table 2 [file 41541_2017_21_MOESM4_ESM.docx]

**Supplementary Table 2. Primers and probes for droplet digital PCR analysis**

| Mutation site | Name^2,3^ | Sequence^1^ |
| --- | --- | --- |
| M-795 | HEX-MP-M795-BHQ | 5’-HEX-AGT CAG CTC ATC ACC TCA ACA-BHQ-3’ |
|  | FAM-ZH-M795-BHQ | 5’-FAM-AGT CAG CTC ATT ACC TCA ACA-BHQ-3’ |
|  | Taq-M795F | 5’-ACA CAC TGT CCA AAT GAC TAC C-3’ |
|  | Taq-M795R | 5’-TAG GAG GGC ACT TGA CTG AA-3’ |
|  |  |  |
| M-3564 | HEX-MP-M3564-BHQ | 5’-HEX-ATA TAT CTT GGA GGA ACA GGC CT-BHQ-3’ |
|  | FAM-ZH-M3564-BHQ | 5’-FAM-ATA TAT CTT GGA AGA ACA GGC CT-BHQ-3’ |
|  | Taq-M3564F | 5’-TTG GGC TCT TTT TCC TCC TT-3’ |
|  | Taq-M3564R | 5’-CCT TCT TAG TGG CAG CAA GC-3’ |
|  |  |  |
| L-533 | HEX-MP-L533-BHQ | 5’-HEX-CAT GGT GCA TGG TCT AAT CTG G-BHQ-3’ |
|  | FAM-ZH-L533-BHQ | 5’-FAM-CAT GGT GTA TGG TCT AAT CTG G-BHQ-3’ |
|  | Taq-L533F | 5’-GCA GGA CTG TTG TTC TTT ACG-3’ |
|  | Taq-L533R | 5’-ACC TAT AAA CCA TCT CCT CTG CT-3’ |
|  |  |  |
| L-3104 | HEX-MP-L3104-BHQ | 5’-HEX-TGC TCA ATG TTT ACC AAG AAA AGG A -BHQ-3’ |
|  | FAM-ZH-L3104-BHQ | 5’-FAM-TGC TCA ATG TTT ACC AGG AAA AGG A-BHQ-3’ |
|  | Taq-L3104F | 5’-GTG GCC GCT GAT CAT TAG G-3’ |
|  | Taq-L3104R | 5’-ATC AAG CTC CCG ATG ACC AT-3’ |
|  |  |  |
| L-3750 | HEX-MP-L3750-BHQ | 5’-HEX-CTC CTT AGC TGC AAT AAT TCA G-BHQ-3’ |
|  | FAM-ZH-L3750-BHQ | 5’-FAM-CTC CTT AGC TGC AAT GAT TCA G-BHQ-3’ |
|  | Taq-L3750F | 5’-GAA GTG GAA ACA CTA GTA GC-3’ |
|  | Taq-L3750R | 5’-TGT AAT GGA GAG TAC ACT GA-3’ |
| ^1^A single nucleotide difference between two probes is underlined. | | |
| ^2^HEX, hexachlorofluorescein; FAM, 6-carboxyfluorescein; BHQ, Black Hole Quencher-1.  ^3^HEX and FAM probes specifically bind to MP-12 and ZH548 sequences, respectively. | | |
